# On Fractioning the Tire Pyrolysis Oil in a Pilot-Scale Distillation Plant under Industrially Relevant Conditions

**DOI:** 10.1021/acs.energyfuels.2c03850

**Published:** 2023-02-06

**Authors:** Juan Daniel Martínez, Alberto Veses, María Soledad Callén, José Manuel López, Tomás García, Ramón Murillo

**Affiliations:** Instituto de Carboquímica (ICB-CSIC), Miguel Luesma Castán 4, 50018 Zaragoza, Spain

## Abstract

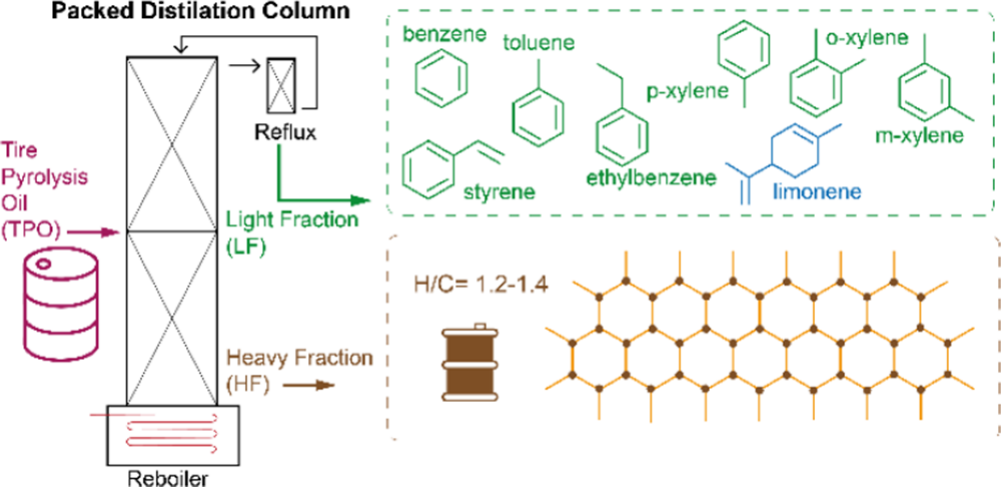

Tire pyrolysis oil (TPO) is one of the most interesting products derived from the pyrolysis of end-of-life tires. Among others, it contains valuable chemicals, such as benzene, toluene, ethylbenzene, and xylene (BTEX), as well as limonene. In order to recover these chemicals, a pilot-scale distillation plant has been designed, erected, and operated using TPO derived from an industrial-scale pyrolysis plant. The distillation facility consists of a packed column (20 kg/h) and is within the fifth technological readiness level. This work describes for the first time the fractioning of the TPO in a continuous operational mode under industrially relevant conditions. For this purpose, different reboiler temperatures (250–290 °C) and reflux ratios (up to 2.4) were preliminarily assessed on the yields and properties of the resulting products: light fraction (LF) and heavy fraction (HF). Thus, the distillation plant is capable of producing 27.0–36.7 and 63.3–73.0 wt % of LF and HF, respectively. The highest BTEX concentration in the LF (55.2 wt %) was found using a reboiler temperature of 250 °C and a reflux ratio of 2.4. Contrarily, the highest limonene concentration (4.9 wt %) in the LF was obtained at 290 °C in the reboiler without reflux. In this sense, the lower the reboiler temperature, the higher the BTEX, and the lower the limonene concentration in the LF. The main results herein obtained serve to gain key insights to operate packed distillation columns using complex and promising hydrocarbons as TPO in order to recover valuable products. In addition, this work provides significant information for optimizing the recovery efficiencies of both BTEX and limonene, as well as their potential applications including that for the resulting HF.

## 1 Introduction

The worldwide forecast production of tires by 2022 is around 2.4 billion units^1^ with the prospect of continuing to grow. This industry uses important and diverse petroleum-derived products, such as synthetic rubber (butyl rubber and styrene-butadiene rubber) and carbon black, among others. Currently, most of the end-of-life tires (ELTs) generated in developed countries are recycled by material and energy recovery practices, while disposal in landfills and stockpiles still being a common practice in developing countries.^2^ For years, ELTs have been used for synthetic turf, sport and children’s playgrounds, molded goods, asphalt and road paving, and civil engineering. In addition, they have been widely used as a supplementary fuel in cement kilns and powerplants. As an example, these practices account for close to 95% of the ELTs generated in Europe.^3^ Although material and energy recovery have played a remarkable role in the sustainable management of ELTs, upcycling technologies have emerged as a new concept to convert post-customer products into high-value chemicals, materials, and fuels.^4^ It refers to upgraded recycling methods that lead to sustainable production and consumption of commodity-like products, which cannot be achieved merely by conventional methods. These methods enable lower-energy pathways and minimal environmental impacts compared with traditional ones.^5^ In this regard, tire manufacturers are increasingly interested in exploring and implementing circular economy strategies for the upcycling of ELTs, aimed at the production of highly technical secondary raw materials (SRMs).

Pyrolysis is a thermochemical process targeting the production of different gaseous, liquid, and solid energy carriers, depending on conditions applied during the process (temperature, residence time of both vapors and solids). In particular, pyrolysis of ELTs is an alternative to conventional material and energy recovery practices, which is attracting renewed attention given its multiple advantages toward a circular economy. An important number of studies containing a high list of valuable references indicate pyrolysis as a conducive environmentally friendly option for ELT utilization while interesting building blocks are produced.^6−8^ This process is regarded as a chemical upcycling pathway since it aims at digging out the embedded value of the ELTs from its main components: both natural and synthetic rubber, and all the carbon blacks used in tire manufacture. In this manner, pyrolysis of ELTs produces tire pyrolysis gas (TPG) and tire pyrolysis oil (TPO) with very interesting characteristics not only as a fuel but also as a chemical pool for producing valuable compounds. Pyrolysis of ELTs also produces a solid faction frequently named for many years and researchers as char, pyrolytic char, or pyrolytic carbon black. However, according to the ASTM D8178 standard, this fraction must be denoted raw recovered carbon black (RRCB), which could be used as a partial substitute or even substitute for virgin carbon black after milling and refining steps as long as its properties match with those required by the final product.

Together with RRCB, TPO is the most important product obtained in the pyrolysis of ELTs when the process is conducted under typical and not very severe conditions. Thus, TPO deserves important attention in order to provide it right impetus following the principles of sustainability and a circular economy. Depending on operational conditions and technology, TPO accounts between 40 and 50 wt % of ELTs. Additionally, TPO exhibits similar characteristics to some fossil fuel oils in terms of heating value, viscosity, and density^9^ and contains an important share of renewable energy given the natural rubber contained in tires that is converted into oil and gas after pyrolysis. For these and other reasons, TPO is considered a competitive alternative to all first-generation bio-fuels in terms of carbon footprint.^10^ In this regard, several studies are found in the literature showing the TPO performance in internal combustion engines as the counterpart of diesel fuel.^10−13^ However, TPO has critical drawbacks such as high sulfur content, low flash point, and high final distillation point, among others, rendering distillation and desulphurization necessary.^14^ TPO is a complex blend of many hydrocarbon families, including among others single-ring aromatics such as benzene, toluene, ethylbenzene, and xylene (BTEX), and in some cases limonene depending on the occurrence of secondary reactions during pyrolysis.^15,16^ Generally speaking, the fewer secondary reactions, the higher the limonene concentration. TPO also comprehends much heavier compounds in the form of tri, tetra, and penta-aromatics, among others. In addition, it contains hydrocarbons containing one sulfur atom in the form of dibenzothiophene and benzonaphthothiophene.^16^

Moreover, TPO plays an outstanding role in the “waste refinery” concept, as it takes advantage of the capacity, technological development, and versatility of conventional petrochemical units to recover a wide range of valuable chemicals.^17^ In this scope, TPO has been subjected to hydrotreating and catalytic cracking processes, having demonstrated the possibility of removing undesired impurities, such as sulfur, nitrogen, oxygen, aromatics, and metals,^18,19^ as well as the production of dry gas, liquefied petroleum gases, naphtha, and light cycle oil.^20^ However, as it was stated before, TPO contains BTEX that are valuable commodities with various and enormous applications in the chemical industry. These compounds are commonly produced from fossil-fueled processes, such as the catalytic reforming (CR), the steam cracking (SC), and the fluid catalytic cracking (FCC). Hence, their recovery from end-of-life products minimizes the reliance on non-renewable sources, providing a significant impetus to a circular economy. Furthermore, the heavier aromatic compounds contained in the TPO are potential feedstock for carbon black production.^21,22^ Typical feedstocks used in this process come from fossil sources and have a very high concentration of aromatics, with a very high H/C ratio, such as coal tar and those derived from the bottoms of the SC and FCC processes.^22,23^ Those feedstocks are frequently used by the furnace black process, which produces practically the entire world’s carbon black, and contributes around 60% of the total manufacturing cost.^24^ Additionally, it is worth mentioning that the global carbon demand for chemicals and derived materials is continuously growing, so the production of waste-based carbon commodities seems to be crucial. By 2050, it is expected to have 1000 Mt of embedded carbon with important participation of end-of-life products as a carbon supplier.^25^ Under this standpoint, ELTs and particularly TPO are expected to play a significant role in the sustainable production of the aforementioned chemical compounds considering the circular economy guidelines.

This work shows the fractioning of TPO by distillation to produce a light fraction (LF) enriched with BTEX and limonene and also a heavy fraction (HF) that encompasses the heavier hydrocarbon compounds. Distillation is probably the most common process in the chemical and petrochemical industries, using tray or packing columns. The latter type supposes a lower pressure drop than the former and is generally shorter in height and diameter. In addition, they offer mechanical simplicity, ease of installation, and the ability to be fabricated in a cost-effective manner from corrosion-resistant materials.^26^ However, the performance of these distillation columns should be tested prior to scaling the process at higher throughput, while identifying the main operational parameters involved when used with alternative and complex hydrocarbons such as TPO. Additionally, it is worth pointing out that most of the experimental data published using packed columns are concerned with binary systems.

Reports on the fractionation of TPO and acquisition of the mentioned chemicals (BTEX, limonene) are rather scarce in the literature. Most of the studies on distillation are found using lab-scale facilities operated at batch mode and aimed at producing fuel-like streams.^14,27,28^ In addition, BTEX and limonene production are mainly focused via catalytic pyrolysis.^29−31^ To the best of the authors’ knowledge, this is the first study showing the continuous fractioning of TPO in a pilot-scale distillation plant using a packed column. The goal of this work is not only to demonstrate the technical feasibility to recover valuable products from TPO by distillation but also to gather key information for future optimization steps, as well as for bringing the process at industrial scales.

## 2 Materials and Methods

### 2.1 TPO Production

TPO was produced in an industrial-scale pyrolysis plant owned by Greenval Technologies based on the single-auger technology, making use of a license of the Spanish Council for Scientific Research (ICB-CSIC). This pyrolysis process is characterized by an intermediate heating of the rubber particles (0.5–2 cm in size) through the walls of the reactor (indirect heating). This plant aligns with the seventh technology readiness level since all its components and systems have been successfully demonstrated in a proper field environment. The mass flow rate of ELTs was 400 kg/h, and the residence time of the feedstock inside the reactor was around 15 min. This technology facilitates the use of a tailored temperature profile throughout the reactor in order to produce quality products. In particular, these operational conditions lead to a higher limonene concentration in the TPO and a very low volatile matter content in the RRCB. The TPG produced during the pyrolysis process is used in situ as fuel to satisfy the energy demanded by the process. Thus, hot gases from TPG combustion are fed in the last section of the reactor in counterflow with the feedstock stream in such a way that the temperature profile inside the reactor decreased from 750 to 500 °C. The heating rate of the ELT particles is around 100 °C/min. Accordingly, the yields of TPG, TPO, and RRCB under these conditions were 20 ± 2, 40 ± 2, and 40 ± 2 wt %, respectively. Once the TPO was discharged from the condenser, it was subjected to filtration and water removal before to be used in the distillation plant. Water in TPO mainly comes from the moisture of the ELTs, which is released after pyrolysis.

### 2.2 Characterization of TPO, LF, and HF

Table 1 shows the methods and analyses conducted on the TPO, and in some cases for both LF and HF. These analyses include elemental composition, higher heating value (HHV), and some key properties, such as density, viscosity, water content, flash point, pH, and total acid number (TAN). TPO and the distillation products were also characterized in terms of the boiling point range by means of simulated distillation (SimDist) using the ASTM D2887 standard. For this purpose, it was used a GC PerkinElmer Clarus 590 GC equipped with a programmable on-column (POC) injector, a wide-range FID detector, and a 10-m Elite-2887 column (0.53 mm ID and 2.65 μm df). An initial oven temperature of 45 °C was maintained for 2 min. A heating rate of 15 °C/min was then implemented to achieve a final oven temperature of 325 °C, which was maintained for 15 min. The carrier gas was He at a constant column flow rate of 7 mL/min. The POC injector was set to programmed mode with a setpoint equal to the oven setpoint temperature plus 5 °C, and the wide-range FID temperature was set at 350 °C. The sample volume injected was 0.5 μL in splitless mode with an autosampler. ASTM D2887 quantitative calibration mixture, containing n-paraffin in the range from C_6_ to C_44_, was injected in order to obtain a correlation curve between the retention time and the boiling point. BTEX and limonene composition were also determined by gas chromatography using the second analysis channel of the same Perkin Elmer Clarus 590. This second channel is equipped with a wide-range FID detector and a 60-m DB-5 ms capillary column (0.25 mm ID and 0.25 μm df). An initial oven temperature of 40 °C was maintained for 1 min, after which a heating rate of 5 °C/min was imposed to reach a final oven temperature of 290 °C. The carrier gas was He at a constant column flow rate of 1 N mL/min. The split/splitless injector and wide-range FID temperatures were 300 and 325 °C, respectively. The sample volume injected was 0.5 μL with an autosampler and a split ratio of 1:30.

**Table 1:** Characterization of TPO

equipment/method	parameter	TPO
Thermo Flash 1112, UNE-EN 15307	carbon (wt %)	88.0
	hydrogen (wt %)	9.8
	nitrogen (wt %)	0.9
	sulfur (wt %)	0.7
from elemental analysis	H/C	1.33
Parr 6400, UNE-EN 15400	HHV (MJ/kg)	42.04
picnometry	density @ 25 °C (g/mL)	0.92
Crison Titromatic, ASTM E203	water content (ppm)	153
Grabner Instruments, ASTM D6460	flash point (°C)	< 25
Mettler Toledo T50	pH (−)	6.4
Mettler Toledo T50	TAN (mgKOH/g)	5.3
simulated distillation (ASTM D2887)	IBP (°C)	69.0
	*T*_50_ (°C)	243.1
	FBP (°C)	513.9
gas chromatography (Perkin Elmer Clarus 590) – FID detector and 60-m DB-5 ms capillary column (0.25 mm ID and 0.25 μm df)	benzene (wt %)	2.1
	toluene (wt %)	6.2
	ethyl-benzene (wt %)	1.0
	(p + m)-xylene (wt %)	5.0
	o-xylene + styrene (wt %)	1.8
	total BTEX (wt %)	16.2
	limonene (wt %)	2.7
gas chromatography (Varian CP-3800) with mass spectrometry detection (Saturn 2200), CP Sil 8 CB capillary column (60 m, 0.25 mm ID, 0.25 μm film thickness)	aromatic compounds (%)	59.9
	PAH (%)	27.2
	naphthenic compounds (%)	6.4
	heterocyclic compounds (%)	3.0
	aliphatic compounds (%)	2.7
	others (%)	0.7

The chemical composition of the TPO was determined using a Varian CP-3800 GC connected to a Saturn 2200 Ion Trap MS. In total, 1 μL of the sample (50 μL diluted to a final volume of 500 μL in a mixture of 1:1 CH_2_Cl_2_/C_2_H_6_O) was injected in the split mode with a ratio of 25:1. A low-bleed capillary column, CP-Sil 8 CB: 5% phenyl, 95% dimethylpolysiloxane (60 m × 0.25 mm i.d. × 0.25 μm film thickness) was used. An initial oven temperature of 40 °C was maintained for 4 min keeping a ramp rate of 4 °C/min until a final temperature of 300 °C for 21 min. The carrier gas was He (BIP (built-in purifier) quality) at a constant column flow of 1 mL/min. The injector, detector, and transfer line temperatures were 280, 200, and 300 °C, respectively. The MS was operated in electron ionization mode within a 35–550 *m*/*z* range and individual compounds were identified by the NIST2011 library. A semiquantification based on the relative area was carried out by using the base peak, and for comparative purposes, the results should be only analyzed among them. A total of 103 compounds identified in the TPO were divided into the following chemical families: aromatic compounds (29 compounds), cyclic or naphthenic compounds (10 compounds), heterocyclic hydrocarbons (6 compounds), polycyclic aromatic hydrocarbons (PAHs) (43 compounds), aliphatic compounds (13 compounds), and others (2 compounds). Each sample was analyzed by duplicate and results were computed as an average, obtaining a relative standard deviation lower than 5% for all the identified families (18% for the others).

### 2.3 Distillation Plant

**Figure 1:**
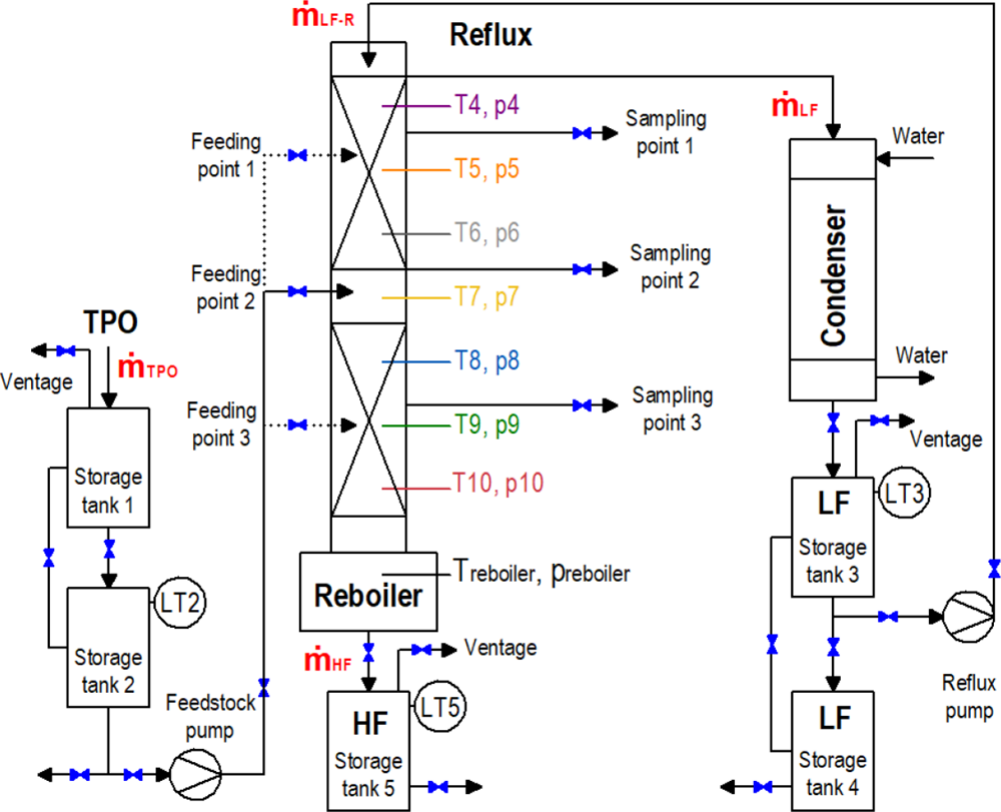
General scheme of the distillation column.

The pilot-scale distillation plant is provided with a single-packed column of 3.5 m of packing height and 4 m of total height. The distillation column considers eight minimum theoretical equilibrium stages, which leads to a height equivalent to a theoretical plate of 0.5 m. The column has 11 cm of internal diameter and uses randomly arranged stainless steel pall rings of 1 inch as packing in which openings are made by folding strips of the surface into the ring. This characteristic increases the free area and improves the liquid distribution conditions inside the column to enhance the mass transfer between the liquid and the gas phases. The bulk density and surface area of these pall rings are 481 kg/m^3^ and 210 m^2^/m^3^, respectively. The packed column works in continuous mode and is capable to process up to 20 kg/h of TPO, which makes the plant to be within the fifth technological readiness level. A general scheme of the plant is depicted in Figure 1.

As it is observed in Figure 1, the TPO is stored in two interconnected tanks (storage tank 1 and storage tank 2) and is pumped toward the column by using a peristaltic pump (feedstock pump) previously calibrated. The TPO level is monitored constantly by a guided wave radar level device (Levelflex FMP50), which is connected with storage tank 2 (LT2). The TPO can be fed to the column by three different points, but only the middle one was used in this work (feeding point 2). The section below this feeding point is known as the stripping section, as the more volatile components are detached from the feedstock; while the section above the feed is commonly denoted as the rectifying section since the concentration of the more volatile components is increased.^32^ Each feeding line is provided with pneumatic valves, which are coupled to the control and acquisition system. In addition, the column is provided with eight thermocouples and pressure transducers located along the distillation column, including those of the reboiler. These signals help to monitor the progress of the distillation process, i.e., transient and steady state, as well as to identify any possible malfunction.

The energy needed for distilling, i.e., to boil the TPO in the bottom of the column, is supplied by the reboiler. Thus, the generated vapors go upward across the column in permanent contact with the downward TPO stream. The low molecular weight vapors become in the LF once they reach the condenser. The reboiler includes an electrical resistance (5 kW) submerged into the TPO. Therefore, it can be accepted that all the energy is efficiently transferred into the TPO. The condenser consists of a shell-and-tube counter-flow heat exchanger and uses tap water to cool down the gas stream that leaves the column. The LF leaves the condenser by gravity, and it is stored in an arrangement of two storage tanks (storage tank 3 and storage tank 4) connected in series. The upper tank serves as a storage for pumping the LF to the distillation column in order to assess the reflux influence during the distillation process. A guided wave radar level device (Levelflex FMP50) is connected to storage tank 3 (LT3). The bottom tank works as a reservoir vessel and sampling. Between these two tanks, there is a feeding line linked to a second peristaltic pump (reflux pump) and the top of the column. Lastly, the higher molecular compounds go downward the distillation column, just below the reboiler. These compounds comprehend the HF, which during operation is collected in storage tank 5 and also provided with a guided wave radar level measurement (Levelflex FMP50, LT5).

### 2.4 Experimental Procedure

Each test at the distillation pilot plant lasts one day, which includes feedstock charging, column heating, distillation experiment, shutdown and cooling, and discharging of LF and HF. Experimental goals and operation mode were varied continuously in order to test several subsystems in terms of stability and reliability during long-term operation. Thus, a typical experiment is described as follows: storage tank 1 is charged with 80 kg of TPO. Afterward, all measuring instruments and ancillary equipment (peristaltic pumps, reboiler, and condenser) are switched on, while pneumatic and hand valves are properly adjusted. This is followed by charging the bottom of the distillation column with TPO up to fully cover the internal electrical resistance (reboiler). Thereafter, the reboiler temperature is adjusted to the established value of the experiment (column heating). The TPO is fed into the distillation column once the reboiler temperature achieves the settled value, and during this work, it was fixed at 20 kg/h in all cases. The reflux pump was switched on after 20–30 min of TPO feeding. Throughout all operations, temperature and pressure signals were recorded and graphed instantaneously. The cooling-down and shut-down of the distillation plant occur in a safe state, i.e., without feeding TPO to the distillation column and without providing power to the reboiler. Finally, HF and LF are collected and weighed in order to know the resulting yields in terms of the reboiler temperature and reflux ratio.

In this work, special attention was paid to the preliminary effect of both reboiler temperature (250–290 °C) and reflux ratio (up to 2.4) on the yields and properties of LF and HF. Neither the TPO nor the reflux was preheated before being fed to the distillation column. This temperature range was selected in order to recover both BTEX compounds and limonene based on lab-scale distillation experiments previously conducted. The reflux ratio (*R*) is defined as the ratio of the liquid mass flow returned to the column divided by the liquid mass flow removed as a product. In other words, it is the ratio between the LF that is introduced again into the distillation column (*ṁ*_LF-R_) and the LF that is collected in the storage tank 3 (*ṁ*_LF_). The reflux ratio in packed distillation columns is usually comprised between 1 and 3.^33,34^ When the reflux ratio is high, the separation efficiency is expected to be high but, at the same time, the operating cost is increased given the power increase of both: reflux pump and reboiler.

## 3 Results and Discussion

### 3.1 TPO Characteristics

Table 1 summarizes the analytical results of TPO. As it was expected, the TPO exhibits not only an important energy density (42.04 MJ/kg) but also a high content of carbon and hydrogen, which leads to a H/C atomic ratio of around 1.33. This value suggests the presence of paraffinic and aromatic compounds.^14^ The contents of sulfur and nitrogen are 0.9 and 0.7, respectively. The oxygen content, determined by difference, is also low (0.6 wt %) and agrees with those found in the literature.^6,7,35^ Aiming toward other applications out of the scope of this work, the aforementioned compounds can be notably decreased by using hydrotreatment processes as shown elsewhere.^18,19^ Similarly, HHV, density, viscosity water content, flash point, pH, and TAN are very similar to data previously published in the literature.^6,7,35^ Among these properties, it is worth paying attention to pH, TAN, and flash point. All of them suggest preventive measures against corrosion, formation of deposits during storage, handling, and final use, as well as flammability hazard.^35^

In addition, a flash point indicates the temperature at which the hydrocarbon forms ignitable vapors at room conditions, providing a general idea about the presence of very light hydrocarbons. In this regard, the concentration of BTEX compounds is 16.2 wt %, while limonene is found to be 2.7 wt %. These compounds strongly depend on the characteristics of the ELTs as well as the pyrolysis conditions. Thus, the higher the natural rubber in the ELTs, the higher the limonene concentration. However, the higher the temperature during pyrolysis, the higher the aromatics and the lower the limonene in the TPO.^15^ The presence of natural rubber also entails an important renewable content in tires, which can vary from 14 to 30 wt % depending on the type of tire (passenger car tire and truck tire) and also the manufacturer company.^2^ Accordingly, the natural rubber ends in both the TPO and TPG after pyrolysis; therefore, the biogenic share in these two fractions is between 17.5–37.5 and 35.0–75.0 wt %, respectively.^36^

Moreover, the GC–MS results reveal that TPO is mainly associated with aromatic compounds (59.9%), as summarized in Table 1. The other family contributing remarkably is PAH (27.2%), followed by naphthenic compounds (6.4%) and limonene being the major one. In addition, the GC–MS results show the presence of heterocyclic (3.0%) and aliphatic compounds (2.7%), among others (0.7%). In the PAH family, naphthalene and substituted napthalenes predominated although other PAHs of higher molecular weight like pyrene, phenanthrene, or even benzo(*a*)anthracene were also present. In the aliphatic compounds, it was possible to distinguish olefinic (1.4%), paraffinic (0.9%), and alkynes compounds (0.5%). This characterization demonstrates that this TPO is a mixture of various compounds comprising light and heavy molecular weight hydrocarbons and agrees with previous reports published elsewhere.^37,38^

**Figure 2:**
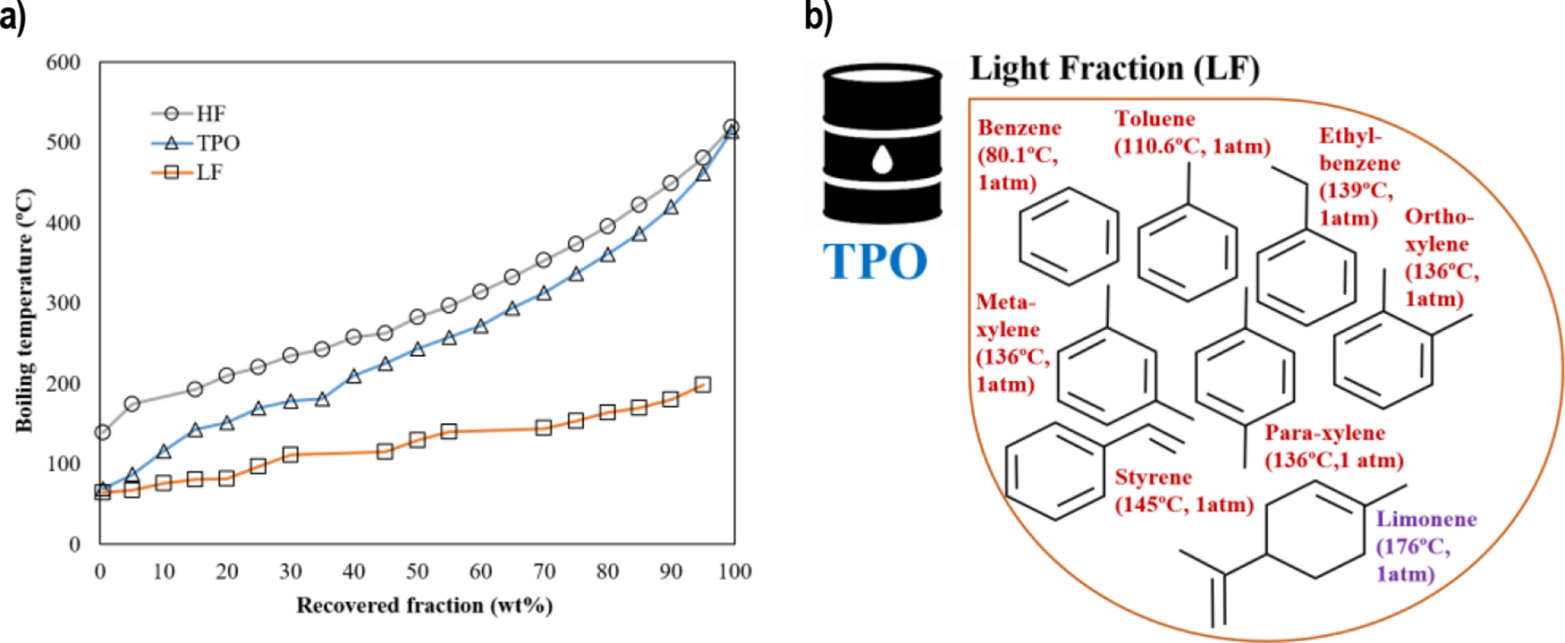
(a) Simulated distillation curve of TPO [LF and HF from the run at 250 °C and 2.4 (reflux ratio)] and (b) schematic representation of the expected compounds in the LF.

Furthermore, Figure 2a shows the SimDist curve of TPO, which provides a general idea not only about the feasibility of fractioning but also about its molecular size and structure. The initial boiling point (IBP) and the final boiling point are 69.0 and 513.9 °C, respectively. Theoretically, the greater the relative volatilities, the easier the separation.^32^ In this regard, Figure 2b shows a schematic representation of the main compounds that could be expected in the LF after distillation, also based on the GC and GC–MS results discussed later. The aforementioned range of boiling temperatures also confirms the complexity of TPO. The distillation temperature at which 50% of the TPO is evaporated (T50) is remarkably high (243.1 °C) and also reflects the unrefined nature of TPO. Finally, it is important to highlight that SimDist is considered a more reliable method than that from atmospheric distillation (AtmDist) using ASTM D86 standard^39^ for determining the boiling range characteristics of hydrocarbon feedstocks spanning a very wide boiling point as TPO. The AtmDist method offers confident data of light petroleum derivates exhibiting an FBP around 254 °C, while SimDist covers a wider boiling range (up to 545 °C).^40^

### 3.2 Distillation Performance

**Figure 3:**
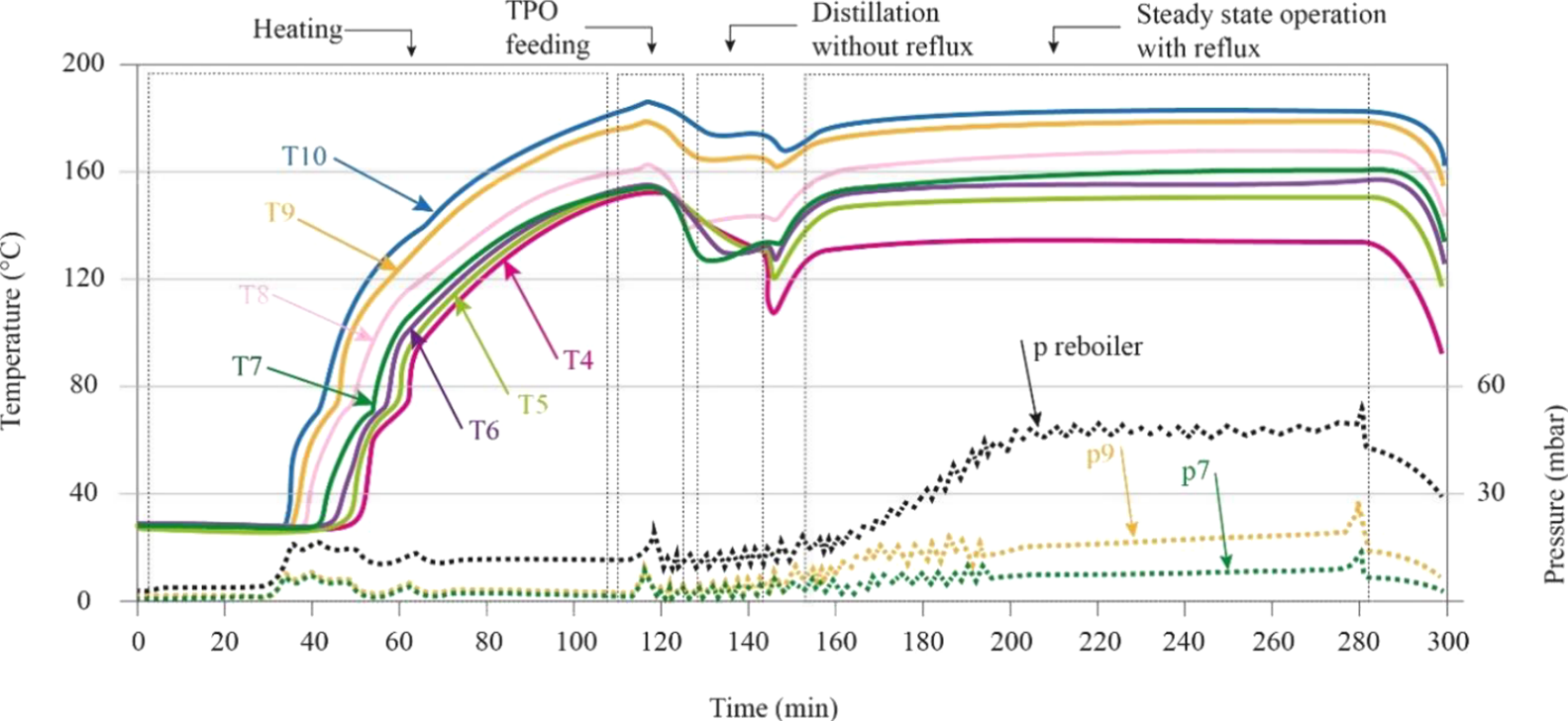
Temperature and pressure profile over the operation time for the experiment at 250 °C and 2.4 (reflux ratio).

Figure 3 depicts an example of temperature at different heights in the column during a typical distillation experiment for the experimental run conducted using a reboiler temperature of 250 °C and a reflux ratio of 2.4. First of all, the column was filled with TPO in order to cover the internal electric resistances and thus avoid possible problems once the reboiler starts to provide energy to the distillation column. This stage takes 20 min. After this, no more TPO is fed, and the column is heated by setting the desired temperature in the reboiler. As an example, it was needed around 120 min to achieve 180 °C just over the reboiler (T10), which agrees with the time reported in other distillation facilities.^41^ Passing over this time, the TPO feeding starts, and after 20 min the reflux pump was switched on once enough LF was generated.

**Figure 4:**
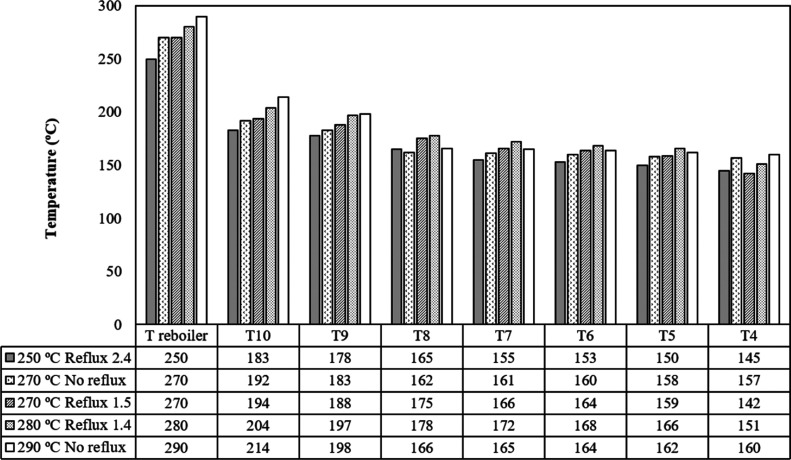
Temperature pattern along the distillation column.

The transition between distillation without reflux and distillation with reflux is very fast (10 min), and the column achieves quickly a pseudo-steady-state operation. The start-up of distillation columns is not a straightforward procedure because of the complex transient responses of hydraulic and thermodynamic variables.^42^ Hence, the pattern exhibited by the distillation column during the start-up period is considered an important achievement since it reveals an adequate control strategy, which is hard to reach in processes using unknown hydrocarbon feedstocks such as TPO. Both bottom and top temperatures reach this pseudo-steady state as the liquid is stripped in the reboiler and as the vapor is rectified, respectively. The supply and distribution of heat at the bottom and throughout the column promote an internal stream circulation, and thus the vapor stream goes upward and liquid downward, without apparent signs of malfunction. As it can be observed in Figure 3, the temperature profile is remarkably flat, indicating a stable distillation process. The temperature along the distillation column decreases from the bottom to the upper part of the column, as expected (T10 > T4), and for this case ranges between 183 and 145 °C, being the reboiler at 250 °C (also see Figure 4). The TPO components are expected to be separated according to their relative boiling points in this temperature range. Hydrocarbons with a low boiling point tend to become enriched in the vapor stream going up the column, while those with a high boiling point are expected to be found in the liquid stream going down the column.

The temperature at the top of the column is the dew point of the overhead product at the column pressure.^43^ This temperature directly relates to the quality of the LF, being the reflux ratio a key parameter on its establishment. In this sense, Figure 4 depicts the temperature profile along the distillation column under steady-state conditions for all the experiments conducted. As it was expected, the packed distillation column is characterized by a decreasing temperature profile from the bottom to the upper part. However, by comparing the experiments with and without reflux, the temperature in the rectifying part of the column (between T8 and T4) does not show the same pattern as that found in the stripping section (at least in the parts covered between Treboiler and T9). The difference in the temperature between T8 and T4 is always lower for those experiments without reflux than those using reflux. For example, the experiments conducted at 270 and 290 °C both with no reflux led to a temperature difference of 5 and 6 °C, respectively; while for the rest of the runs, these differences are higher than 20 °C. These patterns suggest a major capability for recovering hydrocarbons spanning wider boiling temperatures when the reflux is used during distillation. Figure 4 also hints that under the experimental conditions used in this work, the reflux reduces the temperature of the column at the top (see runs at 270 °C with reflux and no reflux).

Likewise, the pressure profiles along the distillation column (preboiler, p9, and p7) are shown in Figure 3. It can be observed that these profiles slowly increased during the heating stage and became stable before TPO was fed, and the reflux was put into operation. As it could be expected, the pressure was higher at the bottom than at the top. In all cases, the pressure in the reboiler was the highest one and increased from 17 to 45 mbar after 40 min. Afterward, the pressure profiles did not exhibit any remarkable change. Therefore, from 210 min of continuous operation, all pressure profiles were kept practically constant suggesting that the steady-state condition had been finally achieved. According to Ray and Das,^43^ the pressure drop does not normally exceed 125 mm of water column per meter of packing height, which in this case means 50 mbar. According to Kister,^44^ the vapor phase tends to channel through the bed at low-pressure drop, leading to poor mass transfer. The pressure drop does not only consider the liquid static head but also some frictional losses through the packing and the phenomena related to expansion, contraction, and changes of direction for both the liquid and the vapor flows. In packed bed distillation columns, the vapor phase is expected to brush the liquid instead of passing through it, which leads to a lower pressure drop than those found in tray distillation units.^45^ Reaching steady-state conditions in continuous operation is a remarkable milestone in distillation facilities aimed at scaling-up.^46^ At this point, all concentrations of the resulting fractions remained constant, and the real influence of the key variables can be determined. After 300 min of continuous operation, a sharp increase in the pressure profile followed by a slow decrease was observed. That indicated that no more TPO was being distilled, in full agreement with the level measures in the tanks.

Both temperature and pressure profiles indicated no accumulation of the TPO while both LF and HF left continuously the distillation column. As a whole, these results also suggest satisfactory gas–liquid mass transfer conditions without any symptoms of instabilities. Therefore, the TPO mass flow rate (20 kg/h), packing (pall rings), reboiler temperature (250–290 °C), and reflux ratios (up to 2.4) used during the experimental campaign, avoided the occurrence of common problems in packed distillation columns such as maldistribution or flooding. Again, this is a remarkable outcome since packed distillation units are hard to ensure suitable liquid distribution throughout the column because of their tendency to move toward the wall and to form channels or preferential paths.^32,41,47^

### 3.3 Yields and Characteristics of the Products

**Figure 5:**
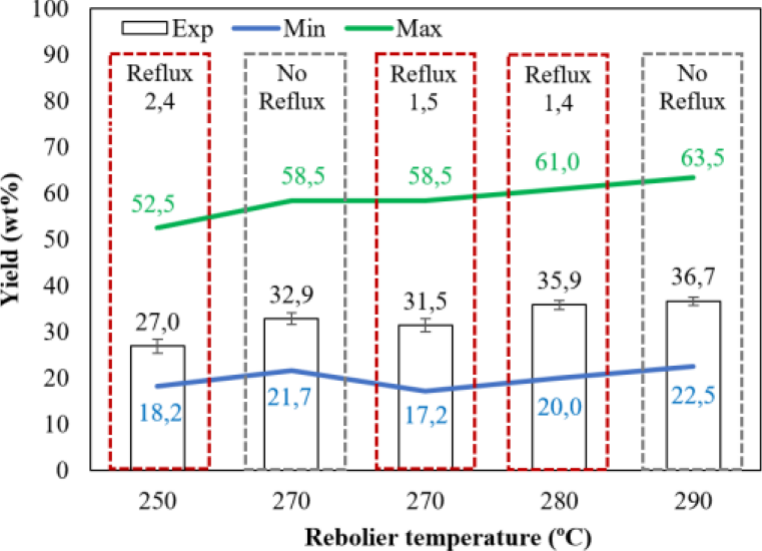
Experimental and hypothetical yields of LF (Min: expected yield using T4 in the SimDist; Max: expected yield using Treboiler in the SimDist).

Depending on both the reboiler temperature (250–290 °C) and the reflux ratio (up to 2.4), the distillation column leads to 27.0–36.7 wt % of LF (Figure 5), while the remaining percent is the HF (63.3–73.0 wt %). The relative error of these yields is lower than 3% in all cases. In addition, it is important to point out that some char particles were observed after each distillation run, which were mainly adhered to the resistance bars of the reboiler. As the amount found was highly lesser than those of the LF and HF, it was not accounted as a product in the experimental campaign conducted in this work. However, it should be considered in long-term trials. Generally speaking, the higher the reboiler temperature, the higher the yield of LF, and the lower the yield of HF, as expected. The LF yields are lower than the hypothetical maximum ones suggested from the SimDist curve (between 52.5 and 63.5 wt %) determined based on the reboiler temperature. However, as it is observed in Figure 4, the packed distillation column exhibits a decreasing temperature profile from the bottom to the top, so the experimental yields also depend on other factors including the temperature at the upper part of the column. Taking into consideration the top column’s temperatures shown in Figure 4, it is also possible to determine the minimum hypothetical LF yield also based on the SimDist curve, as it is observed in Figure 5 (between 17.2 and 22.5 wt %). Likewise, it is worth considering once again that both TPO and reflux were fed to the distillation column at room temperature. Therefore, more liquids must have to circulate by the stripping section and less vapors reached the rectifying zone because at the corresponding feeding points some vapors within the column had to be condensed. The experimental values are between the maximum and minimum hypothetical values, and advise the possibility to improve the yield of the overhead product not only by optimizing the reflux ratio but also by increasing the temperature of the top columns by using preheating strategies for both TPO and reflux, as discussed above.

**Figure 6:**
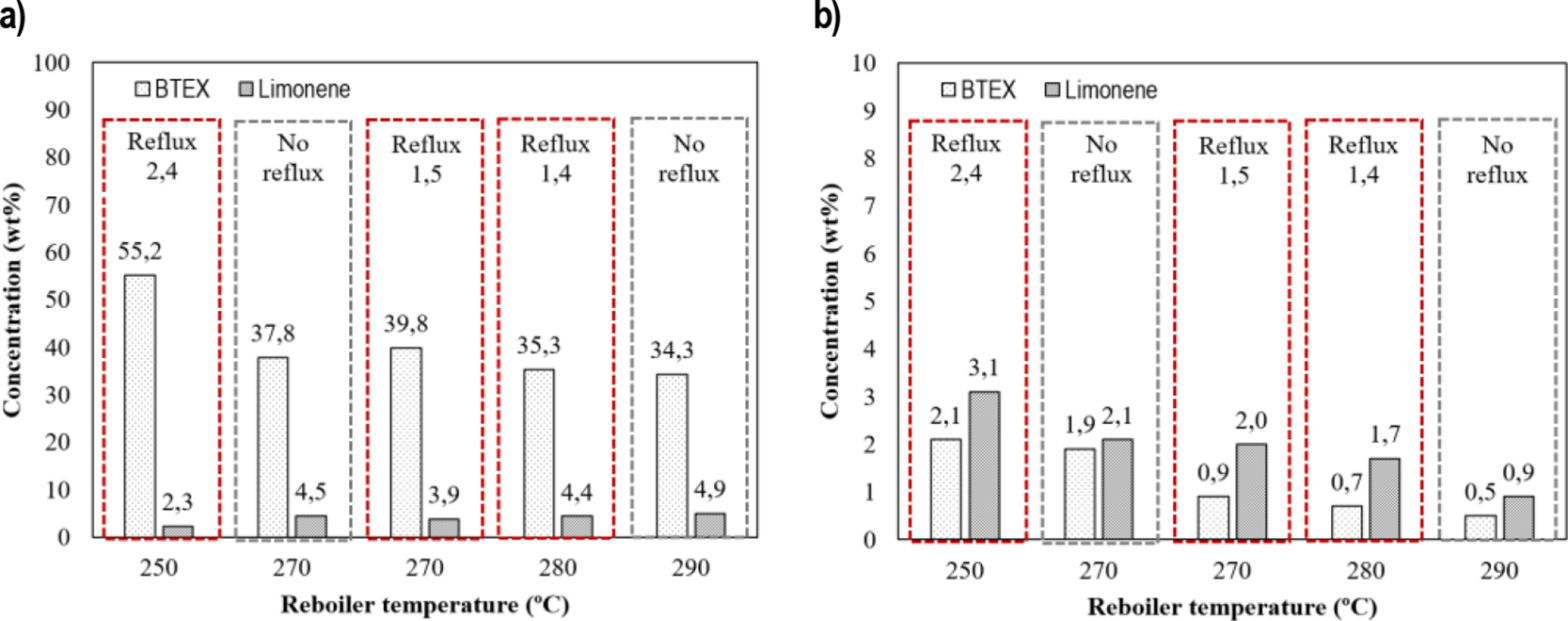
BTEX and limonene concentration: (a) LF and (b) HF.

On the other hand, BTEX and limonene concentrations in both LF and HF are depicted in Figure 6a,b, respectively. It is observed that the highest BTEX concentration in the LF (55.2 wt %) was found using the reboiler temperature of 250 °C and the reflux ratio of 2.4 (Figure 6a). At these conditions, the limonene concentration in the LF was the lowest (2.3 wt %). Conversely, the highest limonene concentration was 4.9 wt %, which was obtained at the highest reboiler temperature (290 °C) without reflux. Although this value is still low, notably, taking into consideration the presence of various isomers and styrene derivatives in the LF and the low concentration of limonene in the TPO, as remarked elsewhere.^48^ In short, these experimental outcomes reveal that the lower the reboiler temperature, the higher the BTEX, and the lower the limonene concentration in the LF. In this sense, the boiling temperature of BTEX is 80.1, 110.6, 139.0, and 136.0 °C, respectively, while the boiling temperature of limonene is higher (176 °C) (see Figure 2b). The BTEX and limonene concentrations in the HF (Figure 6b) hint that the aforementioned compounds are stripped from the HF as the reboiler temperature is increased, as it was expected. However, the higher the reboiler temperature, the higher the concentration of heavier compounds in the LF. Hence, the BTEX compounds are diluted in the LF and, for this reason, decrease with the reboiler temperature, as shown in Figure 6a. As it is observed, this decrease is around 18, 19, and 20% when the reboiler temperature is 270, 280, and 290 °C.

The characterization of the resulting LF and HF is shown in Table 2. As it can be observed, the contents of carbon, hydrogen, nitrogen, and sulfur are practically the same among the LF samples. The H/C ratio and the HHV varied between 1.3 and 1.5, and 40.4 MJ/kg and 41.8 MJ/kg, respectively. Density and water content were comprised between 0.85 g/mL and 0.90 g/mL, and 127 ppm and 144 ppm, respectively. The flash point in all cases was lower than 25 °C, in agreement with the high concentration of very light hydrocarbon compounds such as BTEX. As an example, the SimDist curve of LF obtained at 250 °C and 2.4 (reflux ratio) reveals an IBP and FBP of 64.1 and 198.3 °C, respectively (Figure 2a). The operational conditions used in this work do not either seem to affect significantly the elemental composition and HHV of the HF, although some changes from the structural point of view are expected, as it is shown elsewhere.^14^ However, as compared to the LF, the HF exhibits higher contents of nitrogen (0.7–0.9 wt %) and sulfur (1.2–1.3 wt %), higher density (0.98–1.04 g/mL), higher flash point (>52 °C), and slightly higher water content (180–289 ppm). According to the SimDist curve, the HF obtained at 250 °C and 2.4 (reflux ratio) concentrates the heaviest compounds in the TPO since it exhibits an IBP and FBP of 139.0 and 519.1 °C, respectively (Figure 2a). It is worth mentioning that the boiling point range of TPO (69.0–513.9 °C) involves those from LF and HF, demonstrating once again that continuous TPO fractioning is technically feasible under industrially relevant conditions. All these properties for the HF seem to be very attractive for carbon black production as it is shown elsewhere.^21^ The H/C ratio indicates a good aromatization degree (1.3–1.4). In addition, the resulting flash point (52–64 °C) is enhanced with respect to that from TPO, which suggests less risk during transportation, storage, and handling when the HF is intended to be used in the aforementioned application.^22^

**Table 2:** Characterization of LF and HF

parameter	250 °C, reflux 2.4		270 °C, No reflux		270 °C, reflux 1.5		280 °C, reflux 1.4		290 °C, no reflux	
	LF	HF	LF	HF	LF	HF	LF	HF	LF	HF
carbon (wt %)	87.7	88.3	86.1	86.9	87.5	88.6	87.9	87.7	87.9	87.9
hydrogen (wt %)	11.3	10.2	10.1	9.3	10.4	9.4	10.5	9.3	11.1	9.1
nitrogen (wt %)	0.3	0.7	0.3	0.7	0.3	0.8	0.3	0.7	0.3	0.8
sulfur (wt %)	0.6	1.2	0.6	1.2	0.6	1.2	0.6	1.2	0.7	1.3
H/C	1.5	1.4	1.4	1.3	1.4	1.3	1.4	1.3	1.5	1.2
HHV (MJ/kg)	41.3	42.2	41.5	40.7	41.8	41.9	40.4	41.5	41.0	41.8
density @ 20 °C (g/mL)	0.87	1.04	0.85	1.04	0.90	1.03	0.87	1.03	0.88	1.04
water content (ppm)	147	185	144	185	131	180	143	289	127	191
flash point (°C)	<25	58	<25	52	<25	64	<25	62	<25	62
pH (−)	6.6	6.2	6.6	6.4	6.7	5.9	7.3	5.7	6.5	5.7
TAN (mgKOH/g)	1.74	7.68			1.78	9.55			3.97	9.48
limonene (wt %)	2.3	3.1	4.5	2.1	3.9	2.0	4.4	1.7	4.9	0.9
benzene (wt %)	6.4	0.1	5.3	0.1	5.3	0.0	4.5	0.0	4.5	0.0
toluene (wt %)	23.5	0.5	14.2	0.4	14.5	0.1	11.4	0.1	11.8	0.1
ethyl-benzene (wt %)	3.8	0.3	2.6	0.4	3.0	0.3	4.1	0.2	3.5	0.1
(p + m)-xylene (wt %)	16.2	0.8	10.8	0.7	11.6	0.2	9.4	0.2	9.8	0.1
o-xylene + styrene (wt %)	5.3	0.4	4.9	0.3	5.4	0.3	5.9	0.2	4.7	0.2
total BTEX (wt %)	55.2	2.1	37.8	1.9	39.8	0.9	35.3	0.7	34.3	0.5

**Figure 7:**
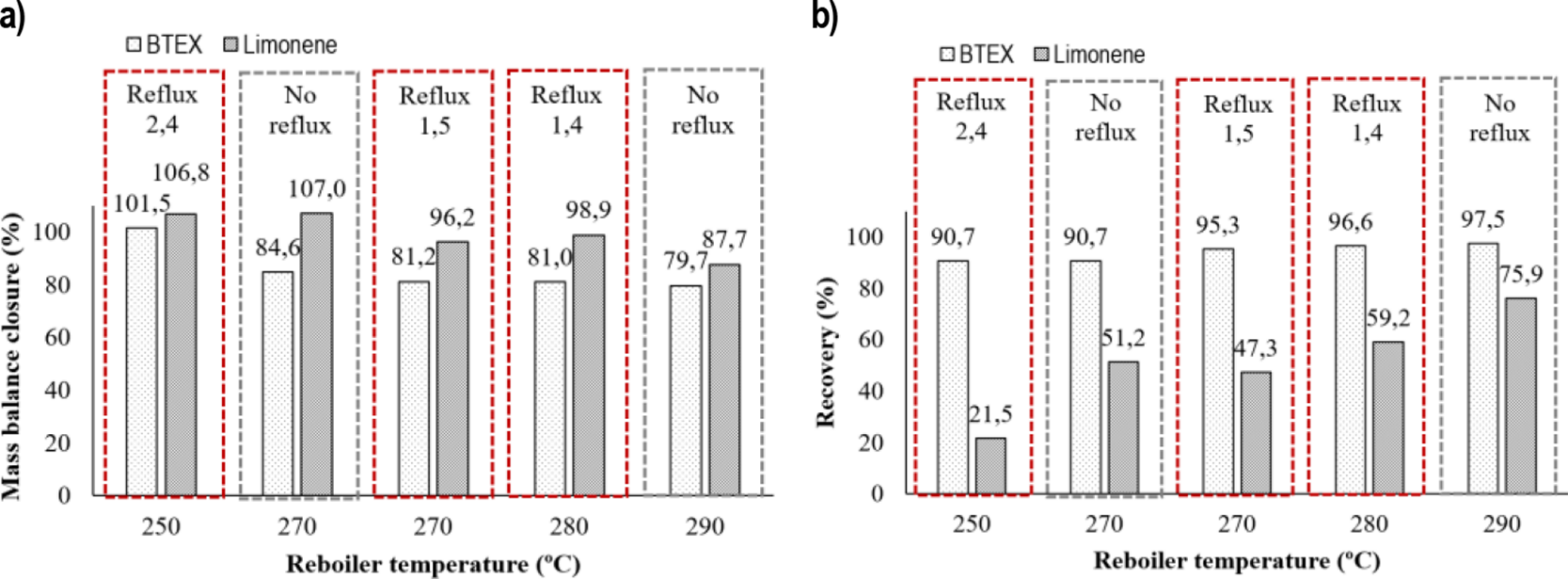
(a) Mass balance closure and (b) recovery efficiency.

The mass balance closure (MBC) of both BTEX and limonene taking into consideration the initial concentrations in the TPO (Table 1) as well as the yields and concentrations of BTEX and limonene found in each experiment (Table 2) is shown in Figure 7a. As it can be observed, the MBC can be regarded as satisfactory (79.7–107.0%) bearing in mind the complexity in the operation of these facilities at pilot scales, as well as the associated error not only in measuring yields but also in determining the concentrations by GC. Similar MBCs were reported elsewhere by using batch distillation and only 500 g of TPO.^49^ The recovery efficiency for both BTEX and limonene in the LF is shown in Figure 7b, and ranges from 90.7 to 97.5%, and from 21.5 to 75.9%, respectively. At first glance, these results agree with the expected behavior regarding the effect of reboiler temperature as a key parameter in the fractioning of TPO and offer an interesting window for future optimization studies paying special attention to the reflux ratio and the temperature of both TPO and reflux prior to being introduced to the distillation column, as discussed above.

In addition, these outcomes suggest that BTEX recovery is easy to reach, as it groups several compounds rather than only one. Generally speaking, BTEX is often used in the production of consumer goods such as adhesives, cosmetics, inks, paints, pharmaceuticals, rubbers, and thinners, making them part of the most abundantly used chemicals worldwide. On the other hand, limonene is a pure compound, requiring more efforts to increase its recovery efficiency by adjusting proper operation conditions in the distillation column. Limonene is a cyclic monoterpene with a large number of industrial applications, including among others, the manufacture of resins and several oxygenate derivatives. It can also be used as a plasticizer precursor in the tire industry,^49^ which supposes a great advantage for the circular economy of tires when a limonene-enriched fraction coming from TPO is used as feedstock in tire manufacture. Limonene recovery can be increased even more after a second distillation, striking positively its value chain.

Finally, the resulting HF does not seem to be very affected by the distillation conditions used in this work. According to the previous characterization, the HF appears to be a prominent alternative feedstock in the furnace process, which produces the most used carbon black by far for rubber manufacture.^50^ Carbon black is listed as one of the top 50 industrial chemicals globally.^21^ It is a fundamental ingredient in tire manufacture since it acts as a reinforcement agent providing strength and durability, as well as improving processing, among others.^51^ This application offers a sustainable approach toward a cleaner route to the production of cost-effective carbon black, herein denoted sustainable carbon back. Hence, the sustainability of the tire industry will be strongly enhanced when this new carbon black is used as a substitute of virgin carbon black in tire manufacture. Based on the above, these results can be regarded as an outstanding breakthrough in TPO fractionation since it reveals the technical feasibility of continuous distillation for recovering value-added compounds.

## 4 Conclusions

The TPO fractionation in continuous mode by distillation was successfully demonstrated in a packed column under industrially relevant conditions. The distillation column under study proved to be capable of recovering value-added hydrocarbons contained in TPO. The temperature and pressure profile along the distillation column were found to be very stable over the operation time and suggest no accumulation of the TPO while both LF and HF left continuously the distillation column. As a whole, these results suggested satisfactory gas–liquid mass transfer conditions under the TPO mass flow rate (20 kg/h), packing (pall rings), reboiler temperature (250–290 °C), and reflux ratios (up to 2.4) used. Using these experimental conditions, the LF was between 27.0 and 36.7 wt %, while the HF was between 63.3 and 73.0 wt %. BTEX compounds and limonene were enriched in the LF and depleted in the HF. The highest BTEX concentration in the LF was 55.2 wt %, when the temperature of the reboiler and the reflux ratio were 250 °C and 2.4, respectively. Conversely, the highest limonene concentration (4.9 wt %) in the LF was obtained at 290 °C without reflux. In this sense, the lower the reboiler temperature, the higher the BTEX, and the lower the limonene concentration in the LF.

The preliminary experimental results found in this work provide an important impetus toward the recovery of value-added compounds from the TPO. In this regard, the products derived from TPO distillation were amenable to be used as SRMs for tire manufacture, realizing an outstanding example of circular economy in the tire domain. Although the ELTs upcycling by pyrolysis has been paid considerable attention in the literature, there are not many studies showing the technical feasibility of producing value-added products using pilot facilities under industrially relevant conditions as the distillation column used in this work. Hence, the outcomes obtained from this work offer notable insights to truly closing the tire loop using pyrolysis and distillation technologies in line with circular economy strategies.
